# Integrated valorization of industrial and municipal biomass wastes for green ammonia production: a circular bioeconomy approach

**DOI:** 10.3389/fchem.2025.1727944

**Published:** 2025-12-04

**Authors:** AbdulAziz AlGhamdi, Syed Ali Ammar Taqvi, Bilal Kazmi, Salman Raza Naqvi

**Affiliations:** 1 Department of Chemical Engineering, College of Engineering, Imam Mohammad Ibn Saud Islamic University (IMSIU), Riyadh, Saudi Arabia; 2 Department of Chemical Engineering, NED University of Engineering and Technology, Karachi, Pakistan; 3 Department of Engineering and Chemical Sciences, Karlstad University, Karlstad, Sweden

**Keywords:** biomass valorization, green ammonia, waste integration, aspen plus modeling, circular bioeconomy

## Abstract

A sustainable production of ammonia using waste biomass is a new milestone to a low-carbon bioeconomy that is circular. This paper defines an integrated Aspen Plus model which integrates the steam gasification of paper mill sludge and municipal solid waste and the Haber-Bosch process to generate carbon-neutral green ammonia. The synthesis of thermochemical conversion and catalytic synthesis was optimized systematically by altering paper mill sludge feed ratio (20:80, 60:40, and 40:60 wt%), steam toward municipal solid waste ratio and pressures in the synthesis. The highest hydrogen yield (H_2_ = 0.4572) and heating value (7.82 MJ/Nm^3^) was obtained in the 60:40 blend at 800 °C and S/B = 0.025, whereas the highest NH_3_ mole fraction in the solution (0.9493) was obtained under 40:60 blend at 500 °C and 250 bar. The addition of cryogenic CO_2_ removal and water gas shift optimization greatly improved the purification of hydrogen and total carbon capture. The innovation of the work consists in the combined modelling structure that converts heterogeneous waste flows into a closed-loop, low-emission system of ammonia production, which has two advantages in the value of waste and the synthesis of renewable fertilizers. The results present an upscale able and ecologically friendly pathway to next-generation production of ammonia, between circular waste management and green chemical production.

## 1 Introduction

Integration of sustainable production in the conventional chemical process industry is revolutionizing the chemical sector for an eco-friendly future (Taqvi et al., 2024). Biomass, being a very versatile resource for green and cleaner production, offers multiple methods and pathways for the production of value-added products through physicochemical, heterogeneous catalysis, biocatalysis, or electrochemical conversion (Seo et al., 2022). These include production of biofuels via Fischer-Tropsch synthesis (Federico et al., 2021), hydrogen (Rodríguez et al., 2022), and syngas, biogas, bio-methanol (Kazmi et al., 2024), ammonia (Luk et al., 2012), and electrochemical reduction of syngas species and carbon capture. Another major limitation of biomass-based systems though is the fact that biomass prices and reliance on a single feedstock may lower the efficiency of the process when such feedstock is limited. This work provides an initial basis for future detailed studies on green ammonia production by implementing multiple biomass sources to address the challenges associated with the shortage of biomass resources and their related issues (Ang et al., 2022). Recent years have witnessed remarkable advancements in integrating renewable feedstocks into conventional chemical manufacturing (Huang et al., 2022). This includes biomass gasification that has become a central thermochemical route to convert solid bio-residues into synthesis gas (syngas), which can subsequently be further converted into fuels and chemicals (Muhammad et al., 2025). Gasification factors, namely temperature, steam-to-biomass ratio (S/B), equivalence ratio, and feedstock characteristics, greatly affect the composition of the syngas that is mostly composed of Hydrogen (H_2_),carbon monoxide (CO), carbon dioxide (CO_2_) and methane (CH_4_). These parameters need to be optimized so as to achieve maximum hydrogen yield and increase downstream effectiveness (Kakavand et al., 2023; Muhammad et al., 2025).

Combining gasification-produced syngas and hydrogen and ammonia synthesis systems is becoming more and more popular as a possible solution to decarbonizing major industrial processes (Shah et al., 2023; Zhou et al., 2022). Tang et al. have shown that a biomass-based ammonia synthesis cycle with Ca-Cu chemical looping has enhanced high CO_2_ emission reductions without compromising thermal efficiency due to the integration of processes (Tang et al., 2022; Tang et al., 2024). Likewise, Hussain et al. conducted comprehensive Aspen Plus modelling of lignocellulosic biomass steam gasification, and found that the temperature and S/B ratio of the gasifier have a significant positive impact on the hydrogen production and cold gas efficiency (Hussain et al., 2023). The results confirm the justification of sensitivity analysis on important operating parameters in biomass-based syngas generation systems.

Syngas purification and CO_2_ separation are vital for efficient hydrogen purification and ammonia production. A hybrid cryogenic-pressure swing adsorption (PSA) setup to capture CO_2_ in syngas was proposed by Berstad et al., and it was demonstrated that the severity of cryogenic conditions has a strong impact on the PSA recovery and total energy use (Berstad et al., 2024). These hybrid systems give realistic foundations in intensifying the processes in the biomass-to-hydrogen routes. The addition of the water-gas shift reaction (WGSR) to the cleaning of the syngas can further increase the hydrogen yield by converting any remaining CO and water to more H_2_ and CO_2_, which can be separated via a cryogenic and PSA unit to be used further or to be sequestered (Muhammad et al., 2025).

Most recent system level evaluations have also focused on the possibility of biomass-based processes to produce net-zero or even net-negative CO_2_ emissions (Rosa et al., 2021). The study by Chmielniak et al. (2024) (showed that negative emission hydrogen production systems could be achieved by coupling biomass gasification with CO_2_ capture, which is why the strategies of CO_2_ control were important in the production of green NH_3_. Martín and Sánchez (2024) also followed this line of reasoning by modeling several biomass-to-ammonia pathways, and they proposed that the performance of integrated designs with efficient CO_2_ capture and energy recovery can be superior to the performance of standard natural gas-based Haber–Bosch processes in terms of carbon intensity and energy efficiency (Martín and Sánchez, 2024). Aspen-Plus is now commonly used to model the steam gasification of heterogeneous wastes (MSW, PMS and biomass), predict the composition of the resulting syngas, and be sensitive to the major operating parameters, including temperature, S/B ratio and equivalence ratio; multiple studies have shown that with meticulous control over S/B and temperature, the H_2_ and CO fractions can be maximized to downstream ammonia synthesis and tar and methane slip minimized (Shafiq et al., 2021). Aspen-Plus has been used to model co-gasification of PMS with woody biomass or MSW to demonstrate positive synergies (improved calorific value and H_2_ yield) as well as challenges brought by inorganic contaminants (ash, Cl, S) that require a strong gas-cleaning step before the Haber Bosch step (Tang et al., 2024). A number of recent papers identify biomass-derived hydrogen (through thermochemical gasification to water-gas shift to CO_2_ removal) as a potentially viable route to decarbonize Haber Bosch ammonia, but note that the high H_2_ purity and low inert (CH_4_, N_2_, Ar) tolerance of the Haber Bosch loop imposes severe syngas polishing (pressure swing adsorption) and integration options with strong impacts on plant energy balances and economics (Martín and Sánchez, 2024; Yan et al., 2024). Recent literature that covers life-cycle and techno-economic studies points to the fact that net-zero or low-carbon NH_3_ with waste feedstocks requires a high level of heat integration between the gasifier, WGS and Haber Bosch units, (a) the fate of CO_2_ (capture, utilization or venting), and (c) small decentralized units are characterized by low Haber Bosch efficiency, whereas (b) large integrated designs are characterised by economies of scale but at high capital density (Martín and Sánchez, 2024; Yan et al., 2024). And lastly, modeling papers explicitly relating Aspen-Plus gasifier outputs to downstream synthesis loops note consistent knowledge gaps that are directly important to your proposed integrated model: accurate tar and trace-contaminant prediction in Aspen models, dynamic behavior of heterogeneous feedstock, realistic modeling of gas-cleaning units (acid gas removal, PSA/membranes) and detailed energy integration with the HaberBosch loop-all of which substantially change the availability of hydrogen, Haber Bosch conversion and the overall CO_2_ balance (Shafiq et al., 2021; Tang et al., 2024).

Altogether, these recent works confirm that the production of H_2_ and ammonia (NH_3_) based on biomass is a promising way to reach carbon neutrality in the process industries. Using diverse biomass feedstocks, optimized gasification and purification, and advanced CO_2_ capture, closed systems can be developed to produce renewable fuels and fertilizers while reducing greenhouse emissions.

The further development of computational tools and process simulations also increases the opportunity to design and optimize such sustainable production pathways and preconditions the success of the further experimental and techno-economic research in this direction. This combined simulation model forms the basis of providing detailed sensitivity analysis of key process variables including steam-to-biomass ratio, gasifier temperature, cryogenic column pressure and ammonia synthesis pressure.

The objective is to assess the trade-offs between H_2_ purity, NH_3_ yield, CO_2_ capture efficiency and the total energy requirement of the process. Through the use of numerous biomass feeds and closed-loop CO_2_ control, this research aim is to play a role in creating a robust, carbon-free route to sustainable ammonia and hydrogen production, which are among the foundations of the new green chemical industry. This paper underscores the relevance of biomass as an effective way of producing ammonia via the Haber-Bosch process, and how heat can also be generated at the same time to obtain energy needs (Kakavand et al., 2023).

## 2 Process development

Aspen Plus V14 was used to develop the presented model; it comprises biomass feedstocks classed as non-conventional components in Aspen Plus properties, according to proximate and ultimate analysis data found in the literature. Besides, Peng-Robinson equation of state (EOS) was used as the base property method to the interaction of parameters and thermodynamic property. Table 1 has a summary of proximate and ultimate analysis of the feedstock. Proximate and ultimate analysis of paper mill sludge (PMS) and municipal solid waste (MSW) demonstrates that the compositions have great differences, which affect the stability of the process. PMS has less fixed carbon (1.23%) than MSW (10.68%), and more ash (59.57% vs. 36.96%). These variations have a significant impact on gasification and the quality of syngas. The presence of more volatile matter in the MSW (52.36% vs. 39.20 in PMS) results in a higher rate of gasification but necessitates a close temperature regulation to avoid the formation of tar. The increased carbon content of MSW (33.96% vs. 16.46% in PMS) leads to greater heating value of the syngas, whereas the different H/C ratios (MSW: 0.189, PMS: 0.099) result in a different H_2_/CO ratio in the product gas. The ash content of the two feedstocks, especially PMS, is high, and thus it requires strong ash handling systems. In order to accommodate these changes, the ratios of blending were selected strategically, the 60:40 PMS: MSW blend is best suited to produce hydrogen and control the ash content, whereas the 40:60 blend is the best suited to produce ammonia, since it has balanced C/H/N ratios. Process stability is maintained through temperature control at 800 °C, careful steam ratio adjustment, and robust gas cleaning system design to handle varying sulfur and ash contents.

**TABLE 1:** Proximate and ultimate analysis of the utilized biomasses in this study (Kartal and Özveren, 2020).

Biomass	Proximate analysis				Ultimate analysis				
	FC	VM	Moisture	Ash	C	H	O	S	N
PMS	1.23	39.20	-	59.57	16.46	1.63	20.22	1.42	0.70
MSW	10.68	52.36	57.30	36.96	33.96	6.41	18.39	1.54	2.74

### 2.1 Process assumptions

The developed model is a steady state, with no accumulation of mass within the system boundaries. The reaction systems are deemed to be equilibrium-based, and reaction kinetics were neglected (Kazmi et al., 2023). The assumption of neglecting reaction kinetics for gasification and instead approaching an equilibrium-based system was made to simplify the limited data on the reaction kinetics of biomass gasification, which may vary with the biomass source. Similarly for water gas shift reactions equilibrium approach was considered. Whereas, for NH_3_, the detailed modelling with reactor kinetics were considered. The processes involved in this model are considered to be isothermal (Muhammad et al., 2023). The nitrogen was considered as a diatomic gas and ammonia (Naqvi et al., 2021), as other nitrogen species in the syngas are only present in trace quantities. Ash and solids were separated entirely from the gaseous fraction (Mehdi et al., 2023) by implementing a component separator due to the unavailability of the particle size distribution of ash content. Overall, the developed system is considered a solid foundation for future work, providing detailed information.

### 2.2 Process description

The proposed process primarily consists of five sections, beginning with the steam gasification of a biomass blend feedstock comprising dried PMS and MSW. The given system integrates the hot and cold streams to manage the cooling and heating operation through networks of heat exchangers with minimal uses of Heater and cooler blocks. Following are the main subsection of the integrated Hydrogen and ammonia production systems:

1. Biomass blend gasification
2. Cryogenic CO_2_ removal
3. Syngas component separation
4. NH_3_ synthesis unit
5. Water gas shift conversion

#### 2.2.1 Biomass blend gasification

The gasification section consists of a decomposer (DECOMP) modeled as a RYield block flowsheet label 101-R, followed by the gasification of decomposed biomass feed in the presence of steam at 800 °C and 1 bar in GASIFIER (102-R). The hot producer gas from the gasification unit is then free from the ash content in SEP block (101-E) which is then used to heat the water through heat exchanger (101-C). The syngas from 101-C is then used further for heat NH_3_ feed to 480 °C through another heat exchanger (102-C). Afterwards it introduced into the syngas cooled to ambient conditions for removal of water in cooler (103-CN) followed by flash separator vessel (104-F) for removal of water and the moisture free syngas.

#### 2.2.2 Cryogenic CO_2_ removal

In Section 2, where the syngas is compressed to 10 bar pressure in double stage compressor (201-J) the compressed gas is sent to a second flash separator (202-F) to further reduce the water content of the gas which is then cooled to 10 °C in chiller (203-CN) the chilled syngas then enter the first cryogenic column (204-E) simulated using RadFrac a block to remove majority of CO_2_ from the syngas the overhead stream from 204-E is the compressed again through compressor (204-J) before entering 2nd Cryogenic Column (205-E). The bottom streams from 204-E and 205-E are mixed through mixer block (206-MX) the purity of total CO_2_ captured from the combine cryogenic columns is approximately 98% wt with a recovery of approximately 92 % wt. The overhead from 205-E which is at a temperature of −98 °C and is compressed to 30 bar pressure in 205-J compressor block raising the temperature to −84 °C. The compressed clean syngas is then heated to 30 °C through the heat exchanger 206-C before entering the Section 3.

#### 2.2.3 Syngas component separation

Starting from the PSA unit-1 (301-F) for separating H_2_ gas from the CO and remaining syngas components. The PSA column is simulated using SEP block flowed by a valve to reduce the pressure to ambient to simulated the rejected off gas. The separated hydrogen stream is split into two by implementing a slitter block (301-SPLT). 60% of the H_2_ is sent to Ammonia Synthesis while the remaining 40% is considered as the product. The Off Gas from 301-F bottom is compressed again through double stage compressor 301-J and enter into the 302-F PSA column where CO rich fraction is separated from the rest leaving mostly CO_2_, traces of CH_4_ and N_2_.

#### 2.2.4 ammonia synthesis unit

The 4^th^ section starts from 400-J compressors where the air is taken from atmosphere to separate nitrogen and oxygen in PSA unit (400-F) modeled similar to the previous PSA units. The N_2_ from 400-F and H2 from 301-SPLT are combined as feed and compressed through two compressors (401-J and 402-J) with intermediate cooler (401-C) to 200 bar pressure. The discharge from 2nd compressor (402-J) is at 215 °C and is cooled using the water stream from flash separating units 104-F and 202-F by implementing the 402-C heat exchangers to 80 °C. this feed stream is the heated to 380 °C in 102-C which is the heat exchanger used for cooling down the syngas. The heated feed stream for ammonia is the passes through splitter and splits the feed into three parts. With each stream entering the series of three adiabatic reactors (404-R, 405-R, and 406-R) simulated as RPlug model. The outlet from 404-R is mixed with 2nd split stream for cooling the 1st reactor outlet and similarly 3rd split stream is mixed with outlet of 2nd reactor before entering the 3rd reactor. The final reactor discharge is then cooled through a series of heat exchanger and chiller (407-C, 408-C and 409-C) the chilled stream carrying the mixture of reactant and products is fed to the 410-F flash separator to separate the Liquid NH_3_ from gaseous fraction consisting mainly of N_2_ and H_2_ the overhead from 410-F is split into recycle stream and H_2_ stream 80% of the 410 Overhead stream is recycled back to the reaction systems while PSA system is implemented to separate out H_2_ from N_2_ which is mixed with product H_2_ obtained from section 2.

#### 2.2.5 Water gas shift conversion

The 5^th^ section consists of water gas shift reaction for conversion of water that was previously obtained from the syngas cooling (104-F and 202-F) and reacting it with CO fraction of syngas that was separated in 302-F. the reaction is carried at 5 bar pressures and 220 °C the reaction system is simulated as equilibrium based system by implementing RGibbs block (501-R) the reactor discharge is then cooled to separate out unreacted water, through flash separation. The H_2_/CO_2_ rich stream is recirculated to the cryogenic separation section to enhance the carbon capture at the same time boosting the hydrogen production and ammonia synthesis.

**FIGURE 1:**
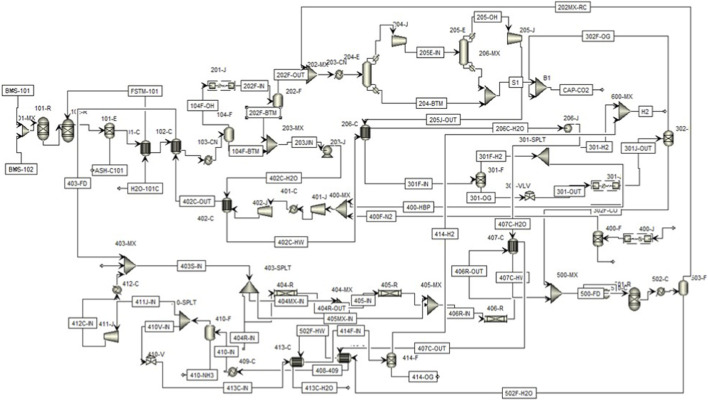
Aspen plus flow scheme for the integrated process utilizing the waste biomass stream for the production of green ammonia.

The offered system proves to be flexible in the handling of the changes in feedstock due to the major design aspects. PMS-MSW blending strategy works as a counter-cyclical to the composition changes, and variable steam-to-biomass ratios (0.025–0.5) help to counter any changes in moisture content. The 800 °C gasification unit gives the gasifier enough thermal power to handle variable feedstock quality to stabilize the production of the syngas without fluctuation in the properties of MSW (organic content 70%–80%), ash content (30%–45%), and heating value (4–8 MJ/kg). The syngas conditioning stages and gas cleaning section further stabilize the process by controlling the changing levels of impurities with the help of temperature and pressure. Figure 1 shows the scheme of the process.

## 3 Optimization approach

This sequential quadratic optimization (SQP) technique was employed, which prioritizes the first optimization of H_2_ production and syngas quality in the gasification stage and subsequent WGSR, followed by optimization of ammonia synthesis using optimized H_2_-rich syngas. The methodology integrates energy efficiency considerations through CO_2_ capture optimization and heat recovery, economic factors through strategic pressure selection and optimization of the steam ratio, and environmental impact through waste valorization and reduction of carbon footprint.

### 3.1 Sensitivity analysis

The optimization strategy adopted a two-stage systematic strategy, which addresses both the gasification and ammonia synthesis subsystems of the integrated process. The fixed conditions of 0.02–0.5 S/B ratio were used by varying the S/B ratio under constant conditions of 800 °C and 2 bar(g) pressure. The gasification temperature of 800 °C was chosen by relying on the best conditions described in the literature with thermochemical transformation of biomass optimized and the tar formation minimized, operation simplicity, and economic viability. The major optimization principles of the gasification section were to obtain as high a hydrogen mole fraction in the syngas as possible and the degree of CO_2_ captured or converted which is an indicator of performance and environmental efficiency.

The second step of the optimization plan was focused on the WGSR and NH_3_ synthesis subsystems, in which the optimization was aimed at fine-tuning the hydrogen enrichment and product formation. The changes in the S/B ratio were also examined in a narrow range to examine how they affected the molar flow rates of hydrogen, ammonia and CO_2_. Also, the temperature changes in the reactor of both the WGSR and the gasifier were analyzed to comprehend the effect they have on the equilibrium composition, conversion efficiency, and selectivity of the products. The analyses allow the determination of the most thermodynamically and operationally feasible conditions to maximize the hydrogen yield and the ammonia production without compromising the sustainability of carbon management.

Based on this, five comprehensive sensitivity analyses were carried out to determine the impact of the important process parameters in the integrated system:

1. Effect of steam-to-biomass ratio on syngas component mole fraction.
2. Effect of gasifier temperature on syngas component mole fraction.
3. Influence of S/B ratio on NH_3_ production, hydrogen generation, and CO_2_ capture.
4. Variation of S/B ratio on hydrogen mole fraction at the WGSR reactor outlet.
5. Influence of WGSR reactor temperature on outlet stream composition.

These sensitivity studies collectively provide a comprehensive understanding of how thermal and compositional variations govern the conversion efficiency, hydrogen enrichment potential, and overall system integration. The resulting parametric trends serve as a foundation for identifying optimal process conditions, supporting a robust and energy-efficient design framework for sustainable ammonia synthesis from biomass-derived syngas.

#### 3.1.1 Effect of steam-to-biomass ratio on syngas component mole fraction

Steam to biomass (S/B) ratio is among the most determining operating parameters of thermochemical gasification process since it determines the degree of heterogeneous and homogeneous reactions occurring in the reactor. The following sensitivity analysis is conducted in the S/B range (0.02–1.0) so as to test its impact on the equilibrium composition of the major syngas constituents of H_2_, CO, CO_2_, H_2_O and CH_4_. With reduced steam ratios, pyrolysis and partial oxidation reactions are favored, which leads to an increased CO and CH_4_ fraction. With a high steam ratio, it offers more involvement of the reactions of steam reforming (C + H_2_O →CO + H_2_) and WGSR (CO + H_2_O →CO_2_ + H_2_) to boost the presence of hydrogen and decrease the level of methane and carbon monoxide. Nevertheless, high addition of steam may result in the dilution effects and low thermal efficiency. Thus, this study is vital in determining the most appropriate S/B ratio that maximizes the yield of H_2_ and the quality of the syngas which has a direct effect on the processes of the downstream catalytic conversion processes of NH_3_ synthesis and Fischer-Tropsch synthesis.

#### 3.1.2 Effect of gasifier temperature on syngas component mole fraction

A very important factor that dictates the equilibrium of the reaction as well as the extent of conversion of carbon in biomass gasifier is the operating temperature of the gasifier. This thermal conditions sensitivity analysis is performed within a temperature of 600 °C–1,000 °C and explores the effect of temperature on relative mole fraction of the key syngas species (H_2_, CO, CO_2_ and CH_4_). The endothermic reforming and Boudouard reactions (C + CO_2_ →2CO) are limited by their kinetics at lower temperatures resulting in incomplete gasification and more methane formation as a result of methanation tendencies. These reactions are thermodynamically preferred as temperature increases, leading to an increase in H_2_ and CO products and a consequent reduction in CH_4_ and CO_2_ concentrations. This analysis is necessary in order to establish the temperature regime that guarantees the optimal carbon conversion efficiency, less tar formation, and quality of syngas that can be used in the next hydrogen extraction and synthesis process. Moreover, the findings inform the choice of suitable materials of the reactor and methods of heat management needed to ensure stable and efficient operation of gasifiers.

#### 3.1.3 Influence of steam-to-biomass ratio on ammonia production, hydrogen generation, and CO_2_ capture

The productivity of the integrated biomass-to-NH_3_ systems is heavily conditioned by the quantity of H_2_ produced in the process of gasification and reforming, and the level of CO_2_ removal or use. This sensitivity analysis is done under the varying S/B ratio within the range of 0.02–0.24 and the resulting effect on the rate of molar flow of ammonia produced, hydrogen generated, and CO_2_ captured in the process network. The selected range is related to a realistic operating range where the contribution of steam input to hydrogen generation through the process of steam reforming and shift reactions does not unduly affect the energy efficiency of the system. When the S/B ratio increases, the hydrogen yield is usually improved, and it subsequently increases ammonia synthesis performance as per the stoichiometric requirement of the Haber Bosch process (N_2_ + 3H_2_→ 2NH_3_). At the same time, an increase in steam level may enhance the CO_2_ production and capture demands, affecting the efficiency of the system to convert carbon and sequest it. This discussion is therefore a mechanistic insight into the effects of steam addition on the concomitant reactions of gasification, reforming, and CO_2_ capture, and helps in optimizing the process of producing hydrogen-rich and carbon-neutral ammonia.

#### 3.1.4 Variation of steam-to-biomass ratio on hydrogen mole fraction at the WGSR reactor outlet

The WGSR is an important intermediate reaction to help to change the H_2_/CO ratio of synthesized syngas, which is required to be used in ammonia production or other processes with high hydrogen demands. The steam entering WGSR reactor in the current system comes as a result of condensing the water collected in the raw syngas stream and thus form a feedback-dependent relationship between the gasification and shift units. This sensitivity analysis examines the effect of the change in the S/B ratio, that is, in the range of 0.02–0.24, on the hydrogen mole fraction at the outlet of the WGSR reactor. Because the presence of steam has a direct impact on the equilibrium concentration of exothermic reaction of the CO conversion, the increase in S/B ratio should cause the reaction to proceed and lead to the rise in the H_2_ generation and CO_2_ formation. But above a certain optimum concentration, more steam could reduce reaction rates and result in dilution of the syngas mixture. This interrelation is significant when designing integrated energy efficient systems, in which internally recovered steam can be used in the most efficient way to attain the desired hydrogen purity to be used in further synthesis reactions.

#### 3.1.5 Influence of WGSR reactor temperature on outlet stream composition

The equilibrium composition of WGSR as well as the rate of reaction is heavily dependent on the temperature of the water-gas shift reactor thus dictating the ultimate percentage of hydrogen in the shifted gas. In order to measure this effect, the reactor temperature was systematically changed between 200 °C and 400 °C and changes in outlet mole fractions of H _2_, CO and CO_2_ were monitored. The WGSR is slightly exothermic (ΔH = −41 kJ mol^-1^) that is, lower temperatures thermodynamically favor the forward reaction to form H_2_ and CO_2_, but higher temperatures enhance the rate of the reaction, but shifts equilibrium towards the reverse. This kinetic/thermodynamic control trade-off requires an optimal temperature range that will guarantee high yield of H_2_ without losing the conversion efficiency. The sensitivity analysis can therefore be of great use in the choice of operating conditions that offer a balance between productivity of hydrogen, heat integration, and the reactor performance of the overall biomass-to-ammonia conversion system.

## 4 Results and discussion

### 4.1 Optimization analysis of steam gasification for a biomass blend of Pulp Mill Sludge and municipal solid waste

**FIGURE 2:**
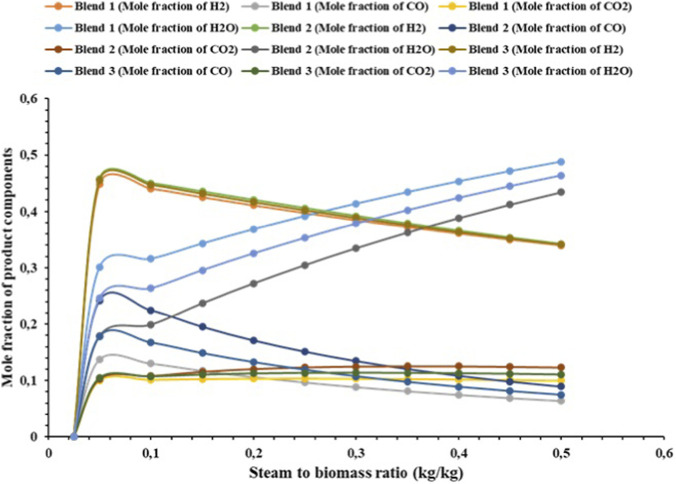
Effect of steam the biomass ratio on the mole fraction of the fractional components of the syngas.

A steam gasification analysis of a hybrid biomass blend of PMS and MSW at different proportions (20 wt.%, 80 wt.%, 40 wt.%, 60 wt.%, 100 wt.%) by varying steam-to-biomass ratios. It can be seen in Figure 2 that the S/B ratio rises between 0.025 and 0.5 in all blends; the mole fractions of major gasification products, namely, H_2_, CO, CO_2_, and CH_4_ are also significantly affected. The maximum mole fraction of the H_2_ (0.4485) is recorded at the lowest steam to biomass ratio (0.025) in the case of the 20 wt.% sludge blends with the 80 wt.% MSW blends and decreases progressively with increase in the steam to biomass ratio, which suggests that there is a potential to obtain maximum H_2_ production with the 20 wt.% sludge blends with the 80 wt.% municipal solid waste blends with lower steam inputs. In the case of blends of 60 wt.% sludge and 40 wt.% municipal solid waste, the H_2_ mole fraction also begins high (0.4572 at a steam-to-biomass ratio of 0.025) but declines with the increase in the steam ratio, indicating that high sludge content may be beneficial to H_2_ production at lower steam conditions. The trends of the 40 wt% PMS and 60 wt% MSW mixture are the same with an initial H_2_ mole fraction of 0.45 at the 0.025 proportion with the same indicating higher H_2_ yields at reduced steam feeds.

**FIGURE 3:**
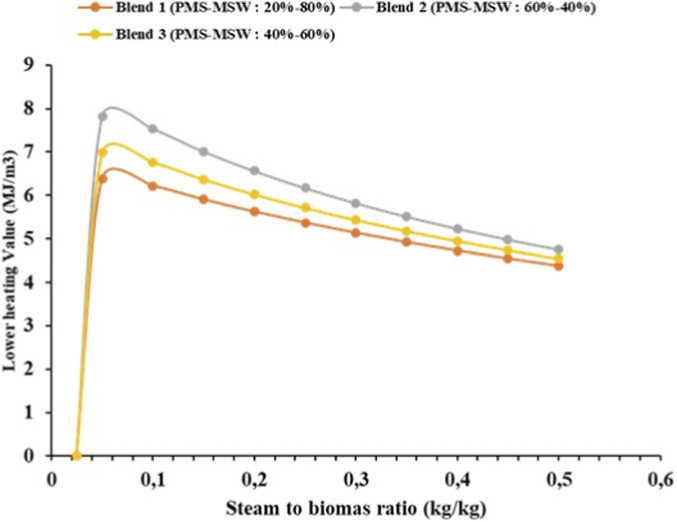
Effect of steam to biomass ratio on the lower heating value of the syngas produced.

The lower heating value (LHV) trend as shown in Figure 3 in the steam gasification of a hybrid biomass blend of PMS and MSW shows a complex relationship with both the blend composition and the steam-to-biomass ratio. For each of the three biomass blend compositions 20 wt% sludge with 80 wt% MSW, 40 wt% sludge with 60 wt% MSW, and 60 wt% sludge with 40 wt% MSW—the LHV generally decreases as the steam-to-biomass ratio increases. This trend is attributed to the increasing steam content, which dilutes the combustible gases and shifts the reaction pathway towards hydrogen production, consequently lowering the LHV. In specific terms, the 20 wt% sludge and 80 wt% MSW blend has a maximum LHV of 6.38 MJ/Nm^3^ at a low steam-to-biomass ratio of 0.025, but this decreases to 4.22 MJ/Nm^3^ as the ratio increases to 0.5. Similarly, for the 40 wt% PMS and 60 wt% MSW blend, the LHV reaches a peak of 6.98 MJ/Nm^3^ at a 0.025 ratio but drops to 4.63 MJ/Nm^3^ at a 0.5 ratio. The 60 wt% PMS and 40 wt% MSW blend displays the highest peak LHV value of 7.82 MJ/Nm^3^ at a 0.025 steam ratio, which gradually reduces to 5.45 MJ/Nm^3^ at a 0.5 ratio.

Overall, the results suggest that a lower S/B ratio (0.025) consistently provides higher H_2_ production across all blends, while higher ratios tend to increase the mole fractions of CO_2_ and decrease CO and CH_4_ fractions. The best configuration in this hybrid gasification process seems to be 60 wt% sludge and 40 wt% municipal solid waste mix in a 0.025 steam-to-biomass ratio, because this ratio results in the highest initial H_2_ yield. Likewise, 60 wt% sludge and 40 wt% municipal solid waste mixture give the highest LHV (7.82 MJ/Nm^3^). This composition contains a large amount of energy, and is thus applicable in processes that need a larger LHV (such as energy production and combustion-based processes, but not hydrogen-oriented synthesis processes, the Haber process). Such setup may be suggested in order to optimize a process in case of the main objective of producing as much hydrogen as possible.

### 4.2 Green ammonia production from hybrid biomass blends using the haber process

**FIGURE 4:**
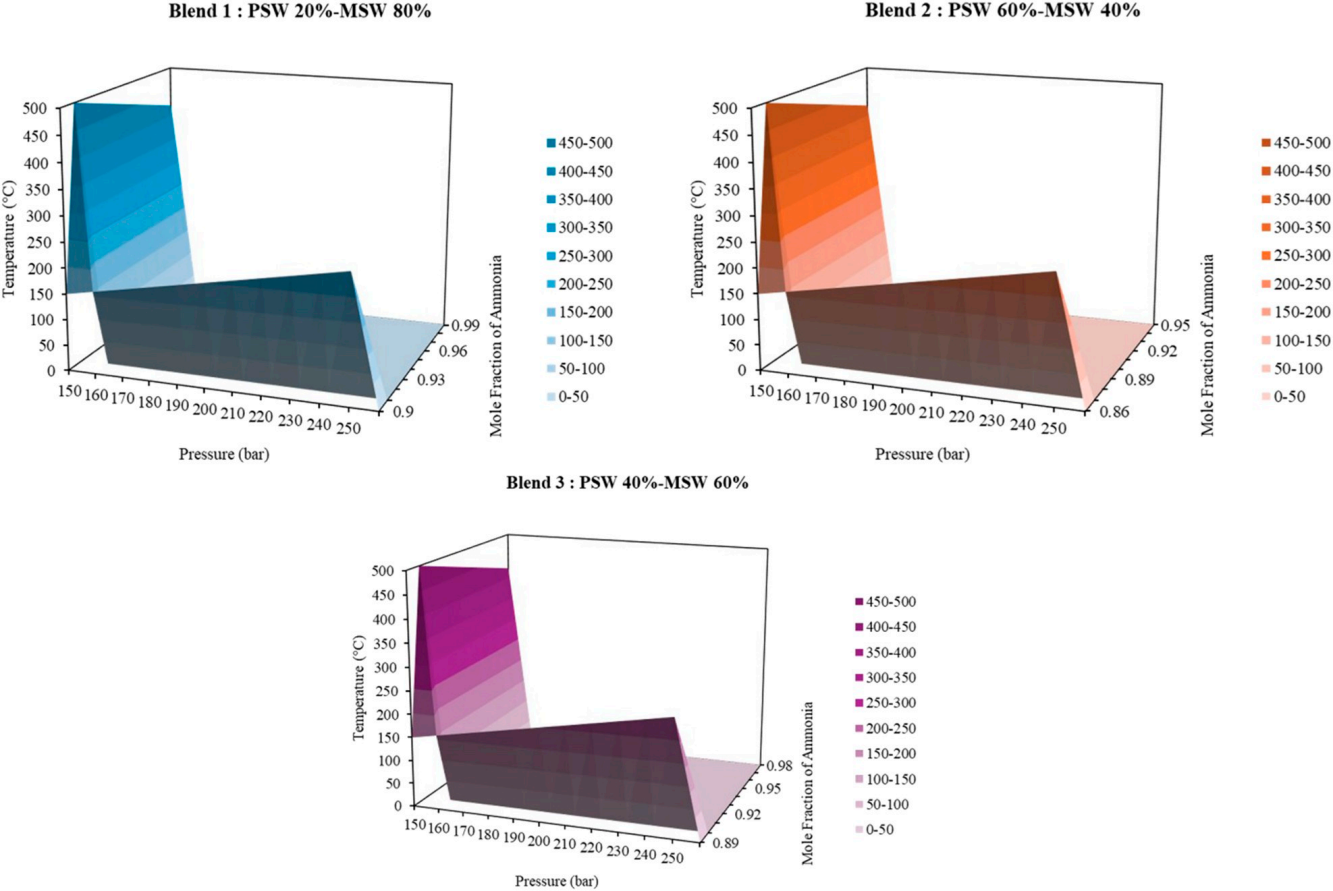
The effect of pressure (150–250 bar) and temperature (0 °C–500 °C) on ammonia mole fraction for different biomass blends of Pulp Mill Sludge (PMS) and Municipal Solid Waste (MSW).

Green ammonia production by the Haber process (with H_2_ generated by the gasification of a hybrid biomass mixture of PMS and MSW) can be maximized by a judicious choice of the biomass mixture as well as tone of the reactor temperature and pressure. Three biomass mixtures (Blend 1 (20 wt% PMS and 80 wt% MSW), Blend 2 (60 wt% PMS and 40 wt% MSW) and Blend 3 (40 wt% PMS and 60 wt% MSW) were examined. In all mixtures, the elevation of reactor temperature (420 °C–500 °C) led to a gradual increase in the NH_3_ mole fraction. Nevertheless, the improvement rate slowed down at temperatures higher than 490 °C (Figure 4). Pressure on the other hand, always enhanced the production of NH_3_ with greater pressures (250 bar) yielding much higher mole fractions of NH_3_. Maximum yields of NH_3_ were at 500 °C and 250 bar with Blend 1 recording maximum mole fraction of NH_3_ of 0.9414, Blend 2 recording 0.9331 and Blend 3 recording 0.9493, being the most efficient blend. More specifically, the Blend 3, which contained 40 wt% PMS and 60 wt% MSW, demonstrated the best NH_3_ yield under all the conditions which is an indication of the Blend 3 providing the best balance between H_2_ generation and NH_3_ formation efficiency. Thus, the most appropriate process optimization model is to operate Blend 3 at 500 °C and 250 bar, as this could generate the maximum fraction of NH_3_ (0.9493) and a more efficient pathway with regard to the conversion of the hybrid biomass feedstock into NH_3_. The high yields of NH_3_ obtained at 500 °C and 250 bar have great energy and environmental implications. Although these operating conditions demand a lot of compression and heating energy, there are a number of efficiency measures that are integrated into the process. The heat exchanger (HTX) system recovers thermal energy, which is designed using the product stream with 450 °C temperature to preheat the feed mixture up to 410 °C and as a result external heating is greatly reduced. There is also the flash separation system which is used at −10 °C and at 150 bar to provide vapour-phase recycle to increase the overall conversion efficiency. The choice of 250 bar pressure though requiring more compression energy is also explained by significant 1.3%–1.7% over 150 bar operation NH_3_ yield increase which leads to less recycling needs and lower energy expenses. On an environmental perspective, the higher carbon footprint of compression energy is compensated by two major aspects, which include the use of waste biomass which would have otherwise led to landfill emissions and the higher conversion rate, which reduces the processing of feedstock. These results point out the significance of balancing between the effectiveness of the process and environmental footprint of sustainable ammonia production systems.

### 4.3 Comparison with the existing literature

Comparative evaluation of recent Aspen Plus-based studies shows that there is a significant progress in integrating biomass gasification with ammonia synthesis to make carbon-neutral fuels. The production of H_2_ was diverse based on the composition of the feedstock, the gasifying agent and the process configuration. The highest H_2_ content (79.8 vol.%) of municipal solid waste was obtained in the present study (Shafiq et al., 2021) through the steam gasification process, which is due to effective reforming of the steam and tar-cracking reactions. Rosha and Ibrahim (2023) established a synergistic effect of combining high-volatile and high-ash feedstocks by showing that co-gasifying pine sawdust with paper-mill sludge increased the H_2_ yield compared to using PMS alone (29.6–38.8 mol%). Mehdi et al. (2023) demonstrated moderate H_2_ yields (34–44 mol%) at different steam-to-biomass ratios, and Dziva et al. (2024) reported over 90 mol% H_2_ using a two-stage sorption enhanced system, which is one of the most hydrogen-selective systems. Contrary to this, the integrated mill residues used by Andersson and Lundgren. (2014) and Arora et al. (2016) produced approximately 42–46 mol% H_2_, which is similar to standard air/steam gasification balances. Hydrogen enrichment was also associated with lower heating values (714 MJ/Nm^3^). However, only a limited number of works, by Dziva et al. (2024) and Arora et al. (2016), have taken the process further to ammonia synthesis, with energy demands of 3640 GJ/t NH_3_, so the gap in the technical maturity between gasification modelling and full-process integration is clear between gasification engineering and the production of green ammonia. The details of the comparative results can be seen in Table 2.

**TABLE 2:** Comparison of Aspen Plus–based biomass gasification studies.

S.No	References	Year	Tech/configuration	Software used	Biomass/blend	H_2_	NH_3_	LHV
1	Shafiq et al. (2021)	2021	Steam gasification	Aspen Plus	Municipal Solid Waste (MSW) (city MSW composition)	79.8 vol% H_2_	N/A	13.1 MJ/Nm^3^
2	Rosha and Ibrahim (2022)	2022	Co-gasification	Aspen Plus	Pine sawdust (woody) co-fed with paper-mill sludge (various CGR)	29.6 mol% H_2_ (single biomass optimal case T = 850 °C, ER = 0.2); ∼38.8 mol% H_2_ at 50% co-gasification	N/A	7.8 MJ/Nm^3^
3	Mehdi et al. (2023)	2023	Steam (and steam/air) gasification	Aspen Plus	Municipal Solid Waste/RDF	34 → 44 mol% H_2_	N/A	LHV reported to vary with conditions
4	Rosha and Ibrahim (2023)	2023	Solid waste gasification + downstream CO_2_ removal (amine) in Aspen Plus	Aspen Plus	Solid waste	∼42.1%	N/A	N/A
5	Dziva et al. (2024)	2024	Two-stage sorption-enhanced gasification (SEG) with *in-situ* CO_2_ capture	ASPEN Plus	Wheat stalk	>90 mol% H_2_	36.4–40.2 GJ/t NH_3_	N/A
6	Andersson and Lundgren (2014)	2014	Integrated biomass gasification	Aspen Plus (process integration)	Pulp and paper residues/biomass integrated with pulp mill	∼42–46 mol%	N/A	N/A
7	Arora et al. (2016)	2016	Small-scale biomass	Aspen Plus	Generic biomass feedstocks	30–90+ mol% H_**2**_	37–40 GJ/t NH_3_	7–14 MJ/Nm^3^
8	This study	2025	Steam gasification	Aspen plus	Blend of paper mill sludge and municipal solid waste	0.4500	0.9493	6.98 MJ/Nm^3^

## 5 Conclusion

This study demonstrates that sustainable ammonia production can be achieved by optimizing steam gasification and the Haber process. A 60 wt.% PMS–40 wt.% MSW blend with a steam-to-biomass ratio of 0.025 yielded the highest hydrogen output (0.4572) and LHV (7.82 MJ/Nm^3^), while the optimal NH_3_ yield was obtained at 500 °C and 250 bar with a 40 wt.% PMS–60 wt.% MSW blend. These results confirm that appropriate feedstock composition and process conditions significantly enhance H_2_ generation and NH_3_ synthesis, offering a sustainable, waste-based alternative to conventional production routes.

## Data Availability

The datasets presented in this study can be found in online repositories. The names of the repository/repositories and accession number(s) can be found in the article/supplementary material.

## Nomenclature

**CO**_**2**_: Carbon dioxide
**CH**_**4**_: Methane
CO: Carbon monoxide
EOS: Equation of state
**H**_**2**_: Hydrogen
HHV: Higher heating value
LHV: Lower heating value
MSW: Municipal solid waste
**NH**_**3**_: Ammonia
PMS: Paper mill sludge
PSA: Pressure swing adsorption
S/B: Steam to biomass ratio
SQP: Sequential quadratic optimization
syngas: Synthetic gas
WGSR: Water gas shift reaction
